# Cardiovascular Reflexes Activity and Their Interaction during Exercise

**DOI:** 10.1155/2015/394183

**Published:** 2015-10-18

**Authors:** Antonio Crisafulli, Elisabetta Marongiu, Shigehiko Ogoh

**Affiliations:** ^1^Department of Medical Sciences, Sports Physiology Lab, University of Cagliari, Via Porcell 4, 09124 Cagliari, Italy; ^2^Department of Biomedical Engineering, Toyo University, 2100 Kujirai, Kawagoe-shi, Saitama 350-8585, Japan

## Abstract

Cardiac output and arterial blood pressure increase during dynamic exercise notwithstanding the exercise-induced vasodilation due to functional sympatholysis. These cardiovascular adjustments are regulated in part by neural reflexes which operate to guarantee adequate oxygen supply and by-products washout of the exercising muscles. Moreover, they maintain adequate perfusion of the vital organs and prevent excessive increments in blood pressure. In this review, we briefly summarize neural reflexes operating during dynamic exercise with particular emphasis on their interaction.

## 1. Hemodynamic Regulation during Dynamic Exercise: General Review and Functions

Physical activities with large muscle mass, such as running, cycling, and rowing, can produce a reduction in systemic vascular resistance (SVR) because of the intense metabolic vasodilatation in the muscle vasculature via functional sympatholysis [1, 2]. This fact constitutes a challenge for the cardiovascular apparatus and it would cause a drop in blood pressure if control mechanisms did not contemporarily augment cardiac output (CO). Thus, the active muscle competes with blood pressure regulation for blood flow. Despite the vasodilation-induced SVR decrease, dynamic exercise in normal subjects is characterized only by a small to moderate increase in mean arterial pressure (MAP) [3–5]. Convincing evidence demonstrates that this fine hemodynamic tuning is determined by the activity of neural mechanisms which control the cardiovascular system and regulate circulation to guarantee adequate oxygen supply and washout of metabolic end-products to exercising muscles. These mechanisms also regulate arterial blood pressure, so that perfusion of the vital organs is reached and blood pressure does not vary excessively.

There are at least three neural mechanisms participating in this cardiovascular regulation: (1) the exercise pressor reflex, (2) the central command, and (3) the arterial baroreflex.

The* medulla* contains the major nuclei that control blood pressure and the cardiovascular system. These nervous circuits are extensively reviewed in other excellent papers [6, 7]. It is believed that the “central command” sets a basal level of sympathetic activity and vagal withdrawal closely related to the intensity of the strain and to motor drive from the motor cortex [8–12]. In this neural mechanism, the cardiovascular control areas located in the medulla are activated by regions of the brain responsible for motor unit recruitment. This basic level of autonomic activation is then modulated by the exercise pressor reflex, which originates from peripheral signals arising from mechano- and metaboreceptors (types III and IV nerve endings within the muscle) that reflexively modulate sympathetic activity taking into account the mechanical and metabolic conditions in the working muscle [12–16]. In detail, it is known that groups III and IV nerve endings excite neurons in the nucleus of the solitary tract (NST) in the medulla. A subset of the NTS neurons activated by these afferents is thought to directly excite neurons of the ventrolateral medulla, which are the primary output for sympathetic activity [6, 7]. This autonomic modulation originating from the central command and the exercise pressor reflex increases HR and enhances myocardial contractility, which together concur in raising CO. Sympathetic stimulation is in turn modulated by baroreflexes, which oppose any mismatch between vascular resistance and CO by controlling muscle vasodilatation and cardiac chronotropism in order to avoid excessive variation in blood pressure [17–19].

Thus, dynamic exercise elicits marked cardiovascular and autonomic adjustments which include increases in CO, MAP, and SVR reduction. This hemodynamic status is regulated by the nervous system by the integration of information coming from the motor cortex (central command), from muscle receptors (exercise pressor reflex), and from receptors in the aortic, carotid, heart, and pulmonary arteries (arterial and cardiopulmonary baroreflexes).

One key point of the functioning of these reflexes is how they interact during dynamic exercise. There is some redundancy between them and neural occlusion can be operative. Moreover, from several observations it appears that both the central command and the exercise pressor reflex can modulate the activity of the baroreflex [19]. In this review, we will briefly summarize the activities of these neural reflexes with particular emphasis on their integration during dynamic exercise.

## 2. Exercise Pressor Reflex

Since the seminal research by Alam and Smirk [20, 21] a great bulk of evidence has demonstrated that metabolic reflex coming from skeletal muscle evokes cardiovascular adjustments during exercise. Subsequently, Coote et al. [22] demonstrated that the muscle pressor reflex could be elicited by ventral root stimulation. Then, McCloskey and Mitchell [8] showed the involvement of group III/IV afferents in this cardiovascular reflex. This reflex is known as the muscle “metaboreflex.” It was later demonstrated that mechanical changes in muscles and tendons can also elicit cardiovascular responses [23]. This reflex has been termed “mechanoreflex.” These two reflexes of muscular origin together constitute the exercise pressor reflex.

It is well established that these two reflexes have their afferent arm in groups III and IV nerve endings within the muscle, with type III nerve afferents mainly acting as mechanoreceptors and type IV as metaboreceptors [24]. It is however important to underline that this classification is not imperative and that both fiber types can act dually as metabo- and mechanoreceptors. Moreover, evidence suggests that mechanoreceptors can be sensitized by metabolites accumulation [25] thereby rendering the specific contribution of mechano- and metaboreceptors to the exercise pressor reflex difficult to evaluate during exercise. These receptors collect information concerning the mechanical and metabolic conditions of contracting muscles and send this piece of information to cardiovascular controlling centers located in the* medulla*, where the information is integrated and elaborated. Then, cardiovascular medullary centers organize the hemodynamic response to exercise taking into account the mechanical and metabolic status of the working muscle [10, 26, 27].

Several substances have been demonstrated to be able to activate the metaboreflex, such as lactic acid, potassium, bradykinin, arachidonic acid products, ATP, deprotonated phosphate, and adenosine [2], whereas the role played by reactive oxygen species is controversial [28]. Moreover, studies with ^31^P nuclear magnetic resonance spectroscopy revealed that the metaboreflex can be activated by decrements in intramuscular pH [29, 30]. These findings are interpreted with the concept that the metaboreflex is activated whenever blood flow to contracting muscles is insufficient to warrant oxygen delivery and/or metabolites washout [13, 31], thereby suggesting that this reflex corrects any possible mismatch between blood flow and metabolism in the muscle. However, there is evidence that in humans the metaboreflex can be active even during mild exercise, when there is sufficient O_2_ delivery to the muscle. In this situation there is no evident mismatch between muscle flow and metabolism, thereby demonstrating the essential role of the metaboreflex in the normal blood pressure response even for light exercise intensities [9]. Therefore, the metaboreflex might be responsible for a tonically active feedback to the cardiovascular control areas which induce cardiovascular changes whenever the muscle metabolism is activated by muscle contractions, even at mild intensities of effort [15, 32, 33].

From a hemodynamic point of view, the typical consequence of metaboreflex recruitment is an increase in MAP [10, 13, 15]. This response is reached by modulating both SVR and CO. However, whilst SVR increase due to sympathetic vasoconstriction is a well-described phenomenon [13, 15, 34], the consequences upon central hemodynamics and CO are less studied and characterized. It is well ascertained that the effect on HR is limited or absent, since studies using the postexercise muscle ischemia method often report very mild or null effects on this parameter [13, 15, 34–39]. However, if the metaboreflex is evoked during exercise by causing muscle ischemia an HR response is evident [40]. The reason for such HR behavior is explained in detail in the* reflex interaction during exercise* paragraph.

In healthy individuals, the metaboreflex can also influence cardiac contractility, preload, and stroke volume (SV) as suggested by recent and past evidence [15, 16, 33, 36, 38, 40–47]. The possibility to recruit the functional reserve of preload and contractility appears crucial since impairment in one or both parameters causes abnormal cardiovascular adjustments to exercise, as observed in situations such as heart failure, spinal cord injured patients, and subjects with diastolic dysfunction [36, 43, 48]. Of note, it has been reported that metaboreflex can induce venoconstriction and splanchnic vasoconstriction, thereby increasing ventricular filling pressure. This phenomenon facilitates venous return and produces a sort of blood volume “centralization” in order to support SV and CO [36, 41, 49]. In particular, a reduction in ventricular filling rate, a measure of diastolic function, has been reported to impair the metaboreflex-induced SV response [36, 38, 49, 50]. Moreover, it has been recently reported that healthy, elderly subjects show an impaired SV response via the metaboreflex as compared to young individuals because of their reduction in cardiac compliance which impaired diastolic filling [43]. Therefore, these results suggest that diastolic capacity is important to achieve a normal hemodynamic response during the metaboreflex.

Thus, the available literature suggests that the hemodynamic response to metaboreflex activation is a highly integrated phenomenon. A complex interplay between HR, cardiac performance, preload, and afterload occurs to achieve, at least in healthy individuals, the normal cardiovascular response to exercise [13, 14, 33, 51].

As concerns the mechanical branch of the exercise pressor reflex, it has been reported that the mechanoreflex can also trigger cardiovascular reflex. Actually, mechanical distortion of type III nerve endings in contracting muscles may substantially increase blood pressure [52, 53]. The mechanoreflex activation has been reported to inhibit cardiac vagal tone which, in turn, causes a rapid and sustained elevation in HR at the beginning of exercise [23]. It should however be kept in mind that, in humans, the mechanoreflex is more difficult to isolate than metaboreflex as muscle contractions, which are needed to recruit the mechanoreflex, are accompanied by both central command and metaboreflex activation, thus rendering the isolation of mechanoreflex from the other two reflexes difficult to achieve. Furthermore, as previously stated, mechanoreceptors can be sensitized by the accumulation of metabolites, which renders the metaboreflex and mechanoreflex contribution difficult to isolate during exercise. For these reasons, research on the mechanoreflex is less abundant than that on metaboreflex and a clear and complete picture of the hemodynamic consequences of pure mechanoreflex activation is lacking. Further studies are warranted to better clarify the role of mechanoreflex in the cardiovascular adjustment to exercise pressor reflex activation.

In summary, from available data it seems that the exercise pressor reflex can adjust all four hemodynamic modulators (i.e., chronotropism, inotropism, cardiac preload, and afterload) to reach the target blood pressure during exercise. However, while the metaboreflex contribution to this reflex is well characterized, less is known about the hemodynamic effects of mechanoreflex activation.

## 3. Central Command

The Nobel Prize winning Krogh and his colleague Lindhard [54] in their early seminal work were the first to propose the concept that the motor cortex could influence the cardiovascular and ventilatory apparatus during exercise. Then, the term “central command” was introduced and it was defined as a “feed-forward mechanism involving parallel activation of motor and cardiovascular centers” [55]. Coherently with the definition, this nervous mechanism does not require any feedback from peripheral muscle. Rather, the central command and the exercise pressor reflex operate in parallel to augment the sympathetic tone during exercise. However, it should be underscored that while central command activation leads to both sympathoactivation and vagal withdrawal [56, 57], this latter effect still has to be demonstrated for the exercise pressor reflex.

It has been demonstrated that central command consists of neural impulses from the motor cortex that irradiate to autonomic neurons in the brain stem and that its activation establishes, at the onset of exercise, a basal level of sympathetic and parasympathetic efferent activity closely linked to the intensity of the exercise performed. Then, this basic autonomic activity is further modulated by the activation of the exercise pressor reflex [8–10, 16]. However, the precise cortical site subserving this mechanism remains unclear. While regions of the higher brain participating in central command activity have been consistently identified (i.e., premotor areas and supplementary motor areas) [58], other brain areas are likely involved in the phenomenon. In particular, studies with neuroimaging and using brain stimulation during surgery have documented that other regions of the brain participate in the cardiovascular regulation during exercise. In detail, cerebellum, insula, anterior cingulate cortex, medial prefrontal cortex, hippocampus, thalamus, and possibly others have all been demonstrated to be potentially involved in this mechanism and all may take part in the circulatory adjustments to exercise [11, 58–63]. Moreover, in recent investigations a key role for the periaqueductal grey (PAG) in the neurocircuitry of central command has been demonstrated, in particular for the lateral and the dorsal lateral PAG. This substance is a functional interface between the forebrain and lower brainstem and it is activated during exercise [59, 63]. In a recent extensive review it has been proposed that PAG fulfils many requirements of a central command center [64].

Whilst it has been demonstrated that exercise pressor reflex activation can regulate the main hemodynamic modulators (i.e., heart rate, cardiac contractility, preload, and afterload; see the* exercise pressor reflex* paragraph), fewer studies have been conducted on the hemodynamic consequences of central command activation, as most of them focused on HR, blood pressure responses, and sympathetic-parasympathetic balance, while less attention has been put on central hemodynamics. It is well ascertained that central command can increase HR and blood pressure by increasing sympathetic and decreasing parasympathetic tone, respectively; however, there are no investigations demonstrating any effect of central command on cardiac contractility, preload, or afterload. This is also because it is difficult to isolate the hemodynamic adjustments due central command activity from those arising from exercise pressor reflex. Further research is warranted to better characterize this topic.

Summing up, central command is a feed-forward mechanism originating from several regions of the brain which modulate autonomic functions on the basis of the motor cortex activation. The typical consequence of its activation is an increase in HR and blood pressure which occurs rapidly at the beginning of exercise.

## 4. Baroreflex

Arterial baroreceptors are located at the medial-adventitial border of blood vessels in the carotid sinus bifurcation and aortic arch. They are pivotal in inducing the rapid adjustments that occur during acute cardiovascular stress via control over HR and peripheral vascular responses to changes in arterial pressure [65, 66]. When arterial blood pressure is elevated or reduced acutely, the baroreceptors are stretched or compressed and this deformation of baroreceptors leads to an increase or decrease in afferent neuronal firing, respectively. These afferent neural responses via baroreceptors result in reflex-mediated systemic neural adjustments with changes in sympathetic and parasympathetic nerve activities, which affect both central (cardiac) and peripheral (vessels) circulation in order to return arterial blood pressure to its original operating pressure point.

### 4.1. Blood Pressure Regulation during Exercise

Since the 1960s, the effect of exercise on the arterial baroreflex function has been reported by many investigators [67–70]. In particular, in earlier studies some investigators questioned the functional role of the arterial baroreflex during exercise [19, 71, 72]. It was believed that the directionally analogous response of HR and arterial blood pressure (increase) to dynamic exercise suggested that the baroreflex was altered or inhibited because the baroreflex-mediated HR responses should be the opposite to change in arterial blood pressure as a negative feedback control system. Therefore, early research suggested that the arterial baroreflex was “switched off” as it was unnecessary for the cardiovascular adjustments to exercise or alternatively that the sensitivity of the reflex was significantly decreased during exercise to increase both HR and arterial blood pressure [19, 70, 72]. Indeed, Iellamo et al. [73, 74] reported that the sensitivity of the cardiac-arterial baroreflex is gradually attenuated from rest to heavy dynamic exercise. Potts et al. [75] were the first to report in humans studies that the full baroreflex stimulus-response curve was well preserved without its maximal sensitivity during increasing exercise workload. These findings suggest that the carotid baroreflex is reset during dynamic exercise and it functionally operates around the exercise-induced increase in arterial blood pressure. Ogoh et al. [76] investigated the physiological mechanism of exercise-induced resetting of carotid baroreflex by using the blockade of sympathetic or parasympathetic nerve activity. In their study, the authors demonstrated that the operating point of the cardiac carotid baroreflex was progressively shifted and relocated in order to regulate the prevailing arterial pressure by vagal withdrawal with reduced sensitivity as compared to its maximum. These inconsistent results are associated with the different methods of analysis. The dynamic analysis of the previous studies (i.e., sequence technique and transfer function analysis) shows only the part of baroreflex function, for example, the baroreflex sensitivity at the operating point but does not allow the determination of the full baroreflex stimulus-response curve in the transition from rest to mild, moderate, and heavy exercise workloads [74, 76]. The upward and rightward shift of the stimulus-response curve to the higher arterial blood pressure and HR allows the baroreflex to operate at the prevailing arterial blood pressure during exercise as effectively as operating at rest, and it also preserves the reflex gain [19, 66, 72, 77]. Further information arises from additional studies showing that this resetting occurs in direct relation to the intensity of effort, without a change in sensitivity [75, 76, 78–80]. Nowadays, exercise-induced “resetting” of the baroreflex function has been well established.

### 4.2. Why Is Baroreflex Resetting Important?

The “resetting” of the arterial baroreflex is essential to evoke and maintain an effective autonomic nervous system modulation and an adequate cardiovascular adjustment to exercise. In exercising dogs, acute denervation of baroreceptors leads to overnormal increase in arterial blood pressure [81]. Similar findings have been reported in humans with surgically denervated carotid baroreceptors. In these subjects, the arterial blood pressure response to exercise is higher than in normal individuals [82, 83]. In addition, when baroreflex activation was counteracted by pharmacologically clamping blood pressure at resting values and preventing the normal exercise-induced increase in arterial blood pressure, a threefold increase in sympathetic nerve activity during handgrip exercise was observed, compared with a control exercise condition [84]. These findings provide proof that the baroreflex acts to finely balance the opposing effects of sympathetic vasoconstriction and metabolic vasodilation, and it also acts to partly restrain the arterial blood pressure response to exercise by buffering activation of the increase in sympathetic activity due to the central command and the exercise pressor reflex.

In other words, if baroreflex function is impaired, then there is an insufficient buffering of the sympathetic tone during exercise. This fact would lead to augmented vasoconstriction and it would lead to a larger increase in blood pressure [19]. Moreover, it might also cause a reduction in muscle blood flow and induce muscle ischemia, thereby contributing to reductions in exercise tolerance [71].

### 4.3. Functional Sympatholysis and Baroreflex

It has been consistently demonstrated that the full expression of sympathetic activation is metabolically inhibited within exercising tissue [85–91]. This phenomenon has been termed “functional sympatholysis.” This metabolic-induced restraint of sympathetic vasoconstriction is also related to the intensity of the effort, as it becomes more evident at harder strains [91–93]. It has been reported that mechanisms for functional sympatholysis are associated with the production of several metabolites, such as nitric oxide [88, 94, 95], adenosine, and prostacyclin [96–98] as well as increases in muscle temperature [99], hypoxia [100], and metabolic acidosis [101]. Interestingly, baroreflex control of blood pressure is well maintained from rest to heavy exercise notwithstanding the attenuation of local vascular response to sympathetic activation in the active muscle. Previously, Keller et al. [102] examined the importance of baroreflex-mediated changes in leg vascular conductance of exercising and nonexercising tissue in the regulation of arterial blood pressure during one-legged knee extension exercise in humans. In this study, carotid baroreflex-mediated reduction in leg vascular conductance to the sympathoexcitation was attenuated in the exercising leg compared with resting condition or the nonexercising leg. This finding indicates the presence of a modulation of sympathetically mediated alterations in leg vascular conductance within the active muscle during exercise. However, despite the attenuation in sympathetic responsiveness (i.e., functional sympatholysis) in the exercising leg, the gains between percentage changes in muscle sympathetic nerve activity and absolute changes in leg vascular conductance were not different in the exercising leg. Importantly, a 3- to 4-fold increase in steady-state leg vascular conductance occurred during exercise in the exercising leg. Therefore, a balance must exist between baroreflex-mediated changes in conductance of a given vascular bed and the influence of exercise-induced attenuation of sympathetic vasoconstriction. Probably, this balance permits a continuous increase in perfusion of the exercising muscle together with a conserved ability of the baroreflex to control vascular conductance which, ultimately, allows maintaining blood pressure during exercise [102]. More importantly, changes in vasomotor, rather than in HR, are the primary targets of the arterial baroreflex in order to regulate arterial blood pressure during mild to heavy dynamic exercise despite a functional sympatholysis [76].

## 5. Reflexes Interaction during Exercise

During exercise, exercise pressor reflex, central command, and baroreflex are all activated and complex interaction occurs between these reflexes. While it is well ascertained that some redundancy and neural occlusion exist between exercise pressor reflex and central command (i.e., their effects do not sum), it is also remarkable that they can all modulate the activity of the other two. The most studied interaction is probably the modulation of baroreflex operated by central command and exercise pressor reflex. In 1990 Rowell and O'Leary [10] proposed a hypothetical scheme of the roles of central command and the exercise pressor reflex in the resetting of the baroreflex during exercise. Subsequently, Raven and colleagues confirmed in a series of experiments this original hypothesis [19, 66, 72, 78]. Thus, it is now well established that both central command and the exercise pressor reflex are involved in the mechanism of baroreflex resetting during exercise. Previous studies that used the vibration technique [103], electrical muscle stimulation [73, 104], partial axillary blockade [105], and partial neuromuscular blockade [106] to manipulate central command in humans demonstrated that selective increase in central command activity relocates the carotid baroreflex stimulus-response curve for both MAP and HR rightward to higher arterial pressures and upward on the response arm without changes in sensitivity. In addition, postexercise muscle ischemia [107], lower positive pressure [108, 109], and medical antishock [110] were used to identify the role of the exercise pressure reflex in exercise-induced baroreflex resetting. An enhanced activation of the exercise pressor reflex relocated the carotid-mean arterial pressure stimulus-response curve upward on the response arm and rightward to higher arterial pressures. However, the exercise pressor reflex only resets the carotid—cardiac stimulus—response curve rightward to operate at higher arterial pressures with no upward resetting. Collectively, these previous investigations identified that both central command and the exercise pressor reflex might reset baroreflex during exercise.

Gallagher et al. [111] assessed the interactive relationship between central command and the exercise pressor reflex for the exercise-induced resetting of carotid baroreflex. In this study, central command and exercise pressure reflex were manipulated by using neuromuscular blockade (vecuronium) and antishock trousers, respectively. Interestingly, exercise-induced baroreflex resetting was greater during the combined enhanced activation of central command and the exercise pressor reflex than during overactivation of either input alone. This finding suggests that central command and the exercise pressor reflex interact. As a consequence, signals from one input facilitate signals from the other, resulting in an accentuated resetting of the baroreflex during exercise. Central command, as a feed-forward mechanism, is likely to be the primary regulator of exercise-induced baroreflex resetting, whereas the exercise pressor reflex operates mainly as a feed-back mechanism. Thus, it exerts a more modulatory role. Furthermore, it seems that both inputs interact and are important for the complete exercise-induced baroreflex resetting [66].

The interaction between reflexes clearly appears during postexercise muscle ischemia (PEMI), a method usually employed to study the cardiovascular effects of metaboreflex activation [15, 33]. During PEMI, there is normally no HR response notwithstanding the activation of exercise pressor reflex and the augmented sympathetic activity. The absence of HR response in this setting is the consequence of the fact that the rise of sympathetic activity due to metaboreflex activation is counteracted by the concomitantly augmented parasympathetic outflow due to the central command deactivation and the concomitant enhanced arterial baroreflex activity that buffers the metaboreflex-mediated increase in MAP [14, 17, 40, 112]. Thus, if the metaboreflex is activated by the PEMI method, the elevated sympathetic activity to sinus node is counteracted by enhanced parasympathetic tone due to the withdrawal of central command and to the sympathetic-buffering effect of baroreflex activation. This fact is not evident when metaboreflex is activated during exercise when central command is operating [40], thereby indicating that central command acts as a modulator of baroreflex activity during exercise.

Along with central command and exercise pressor reflex, cardiopulmonary baroreflex can also modulate arterial baroreflex during exercise. Cardiopulmonary baroreflex plays a pivotal role in maintaining the exercise-induced increase in blood pressure [113, 114]. Moreover, several studies have shown the interaction between carotid and cardiopulmonary baroreflexes. They indicated that unloading of the cardiopulmonary baroreceptors enhanced maximal gain of carotid baroreflex function at rest and during exercise [109, 115–119]. Interestingly, alteration in cardiopulmonary baroreceptor load during dynamic exercise affects not only the prevailing exercise-induced arterial blood pressure, but also the resetting of the arterial baroreflex [120–122].

Ogoh et al. [122] increased central blood volume (cardiopulmonary baroreceptor load) by increasing pedal frequency to enhance the muscle pump at the same amount of central command. Then, they demonstrated that the magnitude of exercise-induced increases in arterial blood pressure was reduced and carotid baroreflex reset leftward and downward during dynamic exercise. Moreover, Volianitis et al. [120] reported that when leg cycling was added to arm-cranking exercise, arterial blood pressure was reduced below that of arm exercise alone and resulted in relocation of the operating point of the carotid baroreflex-MAP curve to the lower arterial blood pressure despite greater activation of central command and the exercise pressor reflex. These findings suggest that input from cardiopulmonary baroreceptors can influence arterial baroreflex control during exercise. In particular, cardiopulmonary baroreflex is associated with the locus of the operating point of the baroreflex-mean arterial pressure curve. Collectively, the cardiopulmonary baroreflex also resets during physical activity to operate around the exercise-induced increase in central blood volume without a change in reflex sensitivity [123]. Therefore, these results indicate that the cardiopulmonary baroreflex plays an important role in baroreflex resetting during exercise and it operates together with central command and the exercise pressor reflex.

Interaction has also been demonstrated between central command and the exercise pressor reflex. Indeed, some evidence suggests that input from types III and IV muscle afference modulates the central command activity and exerts an inhibitory effect on central motor drive. Furthermore, these signals may influence the perception of effort [124]. In detail, it has been demonstrated that attenuation of somatosensory signals from the muscle obtained with epidural anesthesia, which reduced afferent input, resulted in an increase in central command activity. However, HR and blood pressure responses were attenuated as compared to a normal exercise, thereby suggesting that afferent feedback from the muscle is essential in normal cardiovascular adjustments to exercise [9, 124, 125]. Therefore, it seems that central command cannot work properly without adequate feedback from peripheral muscle and that, at the same time, this feedback limits central command and motor drive. However, this is quite a complex issue and further research is warranted to better clarify the complex interaction between central command and exercise pressor reflex.

**Figure 1:**
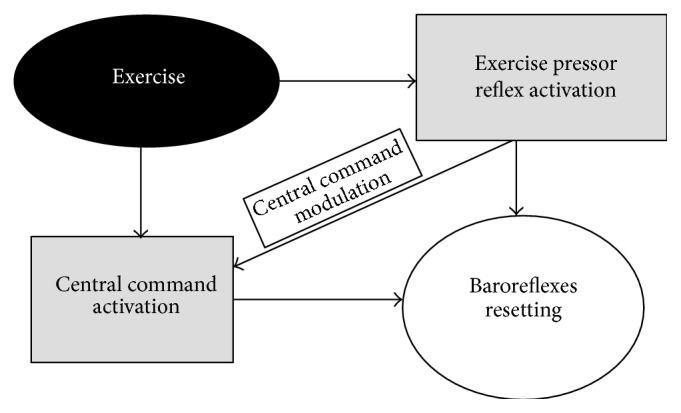
Interactions between the three main neural reflexes operating during exercise. See text for more details.

Figure 1 depicts the various interactions between reflexes which are supposed to be operative during exercise.

## 6. Conclusions

In summary, cardiovascular regulation during exercise is reached through the contemporary integration and interaction between input arising from motor cortex, skeletal muscle receptors, and arterial baroreceptors. While it is well ascertained that baroreflex activity is modulated by both central command and exercise pressor reflex, less is known about the interaction between central command and exercise pressor reflex. Further research in this field is warranted.
